# Proteomic Analysis of NULISA™ plasma analytes and Associations with Neurocognitive measures and Incident Dementia in older adults

**DOI:** 10.1002/alz70862_110194

**Published:** 2025-12-23

**Authors:** Joshua L Gills, Hubert Leo, Omonigho M Bubu

**Affiliations:** ^1^ NYU Grossman School of Medicine, New York, NY USA; ^2^ New York University Langone Health, New York City, NY USA

## Abstract

**Background:**

Neurodegenerative diseases, including Alzheimer's disease (AD), are characterized by progressive cognitive decline, with plasma biomarkers potentially playing a key role in early detection and risk assessment. The Nucleic Acid–Linked Immuno‐Sandwich Assay (NULISA™) offers high sensitivity for measuring low‐abundance proteins associated with AD pathology, including Aβ42/Aβ40, *p*‐tau isoforms, NfL, and GFAP. We examined the associations between NULISA™‐quantified plasma biomarkers and cognitive performance and investigated their predictive value for incident dementia in older adults.

**Method:**

This study utilized data from 116 ARIC cohort participants with available plasma samples. Plasma biomarkers were quantified using the NULISA™ CNS 126 protein platform. ATN & I biomarkers included: A (Aβ proteinopathy) – Aβ‐42, Aβ‐40, and Aβ‐42/40; T (phosphorylated and secreted AD tau) ‐ *p*‐tau217, *p*‐tau181, *p*‐tau231 and their ratios with Aβ‐42; N (neuropil injury) – NfL and Total Tau; and I (inflammation) – GFAP. Cognitive performance was assessed using global and domain‐specific neurocognitive tests, and incident dementia cases were identified through longitudinal follow‐up. Linear regression and Cox proportional hazards models were used to examine biomarker associations with baseline cognition and dementia risk, adjusting for age, sex, APOE ε4 status, education, and glomerular filtration rate. FWE correction was utilized for multiple comparison adjustments.

**Result:**

The 116 participants were on average 74.3 **±** 4.7 years old, 88.8% were cognitively normal, 29.7% were APOE4 carriers, and overweight (BMI=29.7**±**5.5 kg/m^2). Of the NULISA Alamar 126 proteins, Growth differentiation factor 15 was the only protein associated with Incident Dementia (β = 4.7, *p* < 0.001). Antigen CD63 (β = ‐0.43, *p* < 0.001) and Eotaxin CCL11 (β = ‐0.37, *p* < 0.001) were associated with global cognition. Growth Regulated Alpha Protein CXCL1 was associated with executive function (β = ‐0.33, *p* < 0.001). No significant relationships were identified between NULISA Alamar 126 proteins and language or memory scores.

**Conclusion:**

Proteins subserving functions related to synaptic vesicle exocytosis and inflammation were associated with cognition and incident dementia in the ARIC sample, but power is limited to evaluate the full range of proteins. Future investigations should increase the sample size to identify other novel proteomic pathways related to cognition and incident dementia.

